# Prognostic factors and treatment outcomes in patients with non-ampullary small bowel adenocarcinoma: Long-term analysis

**DOI:** 10.1097/MD.0000000000015381

**Published:** 2019-04-26

**Authors:** Jiangfang Tian, Jiewei Liu, Chunhong Guo, Xi Yang, Yu Yang, Hongfeng Gou, Meng Qiu, Dan Cao

**Affiliations:** a Department of Abdominal Oncology, Cancer Center; b Lung Cancer Center, West China Hospital, Sichuan University, Chengdu 610041, Sichuan Province, China.

**Keywords:** Clinical characteristics, prognosis, small bowel adenocarcinoma (SBA), treatment

## Abstract

Small bowel adenocarcinoma (SBA) is a relatively rare malignancy in gastrointestinal tumors. In addition, the difficulty of early diagnosis, its poor prognosis compared to large bowel adenocarcinoma, and inadequate treatment experiences due to lack of prospective randomized trials make it necessary to explore the characteristics of the disease for early diagnosis and treatment.

Patients diagnosed with primary malignant tumor of small intestine in West China Hospital of Sichuan University between January 2001 and 2013 were reviewed retrospectively. A total of 208 patients with SBA were selected and 160 patients with duodenal periampullary tumor were excluded. Forty-two cases of patients were finally enrolled for statistical analysis as 6 patients were lost of follow-up. The clinical characteristics, the response to treatment and their overall survival (OS) time were reviewed and analyzed.

Of the 42 patients, 11 (26.2%) primary tumors were originated from duodenum, 29 (69.0%) from jejunum, and 2 (4.8%) from ileum. All patients (64.3% male; median age, 54.7 years) included in this study underwent primary resection of the tumor to confirm final diagnosis. Three-year survival rate is 21% and 5-year survival rate is 9%. Median OS were 24.2 months (95% CI: 4.0–72.0). The univariate predictors for prognosis of SBA were as follows: age (*P* = .021), severe intestinal symptoms at first diagnosis (*P* < .001), T4 of tumor stage (*P* = .011), tumor size (*P* = .004), relatively late clinical stage (*P* < .001), peritoneal metastasis (*P* < .001), and no chemotherapy (*P* = .011). The multivariate predictors for poor prognosis were age of more than 60 years old (*P* = .035), intestinal obstruction or perforation at first diagnosis (*P* = .026), relatively late clinical stage (*P* = .000), and no chemotherapy (*P* = .027).

SBA was a relatively rare malignancy that was difficult for early diagnosis and treatment. Intestinal obstruction was the common clinical manifestation at first diagnosis, with a tendency of early peritoneal metastasis. Precaution of the disease in early phase, radical resection of the primary tumor while resectable, followed with in-time chemotherapy might improve prognosis and survival of patients with SBA.

## 1 Introduction

Although the small intestine covers about 70% to 80% length of the total gastrointestinal tract and 90% of the total surface area, small intestine cancer incidence rate is far lower than gastric and colorectal cancer (CRC), accounting for 1% to 3% of all gastrointestinal tumors.^[1–3]^ According to the United States National Cancer Database, the incidence of all small bowel cancers in the USA rose from 11.8 cases/million persons in 1973 to 22.7 cases/million persons in 2004.^[4]^ The annual report of the Korea central cancer registry stated that 449 new cases of small bowel adenocarcinoma (SBA) occurred in 2002.^[5]^ Adenocarcinoma is the most common histopathological subtype, accounting for around 40% of all cancers of the small bowel,^[4,6]^ followed by carcinoid tumors, lymphomas, and sarcomas.^[7,8]^ Most SBA originate in the duodenum followed by jejunum then ileum with about 10% having an unknown origin. Adenocarcinoma of the ampulla of Vater and the periampullary region are generally considered separately as cancers arisen from this region might also be pancreatic or cholangetic originated and have vastly different origins, methods of diagnosis, surgical procedures, and poor prognosis.

Because of its rarity, the deep location in small intestine, and its atypical symptoms that might be confused with ordinary malfunction of gastrointestinal diseases, early diagnosis was usually not easy. The prognosis of SBA was between gastric cancer and large bowel adenocarcinoma, with a 5-year OS rate ranging from 14% to 33%.^[9]^ Surgical resection is the mainstay for treatment of SBA.^[10]^ Whether adjuvant chemotherapy after the R0 resection of tumor improves survival, or which first line chemotherapy regimen is recommended for advanced unresectable SBA still remains unclear. The reason for that is because the rarity and sporadic occurrence of the disease makes it difficult for large-sample, prospective randomized trials to take place. Retrospective researches and small-sample phase II trials all around the world are still the main source for the knowledge of the disease.

In the past decades, most researches for SBA were mainly from European, Americans, and Korean. Recently, a retrospective research from China reviewed 119 cases of patients with SBA from 2001 to 2011 with the most frequent location as duodenum including adenocarcinoma of the ampulla of Vater. Through one factor analysis of variance, depth, node involvement, palliative surgery, and the site of tumor are associated with a poor prognosis.^[11]^ However, as adenocarcinoma of the ampulla of Vater differs vastly from SBA, the epidemiology, clinical characteristics, the response to treatment, and prognosis in Chinese patients with SBA (excluding cancer of the ampulla of Vater) were not known very well. Based on that situation, we collected data of patients diagnosed with SBA in West China Hospital of Sichuan University from the year 2001 to 2013, in order to get a better understanding of their clinical characteristics and factors that might be influential to prognosis, whose information might be helpful for a better understanding of SBA, especially in Chinese patients.

## 2 Patients and methods

Patients diagnosed with primary malignant tumor of small intestine in West China Hospital of Sichuan University between January 2001 and 2013 were reviewed retrospectively, among which 208 adenocarcinoma of small intestine was further selected and analyzed. All patients included in this review have undergone surgical resection of the primary site, confirming the pathological diagnosis of adenocarcinoma. One hundred sixty patients with duodenal periampullary tumor or ampula of vater tumor were excluded, as cancers arisen from this region might also be pancreatic or cholangetic originated, which made the final number of adenocarcinoma from other parts of small intestine as 48 cases. Among those 48 cases, 6 cases were excluded because of loss of follow-up, leaving 42 cases for analysis of clinical data, including clinical presentation, diagnostic method, pathological staging, treatment and follow-up data. The tumor-node-metastasis (TNM) stage of the 6th American Joint Committee on cancer staging was used. The World Health Organization standard grading system with its four categories (well differentiated, moderately differentiated, poorly differentiated, and undifferentiated) was used for the histologic grades. This study was approved by Ethics Committee of West China Hospital, Sichuan University. All patients provided verbal informed consent prior to study enrollment.

## 3 Statistical methods

The frequencies and descriptive statistics of the demographic and clinical variables were determined. Chi-square and Fisher exact were used for categorical variables and Student *t* tests were used for the continuous and ordinal variables, as appropriate. The OS time was calculated from the date of diagnosis to the date of death or the last known follow-up. The survival time was estimated using Kaplan–Meier methods. The log-rank test was used to compare the survival curve. The univariate and multivariate predictors of OS were determined by the Cox proportional hazard model. Rate ratios and their 95% confidence internals (CIs) were calculated. The SPSS program for Windows (version 11.0; SPSS, Inc., Chicago, IL) was used for analysis.

## 4 Results

### 4.1 Baseline characteristics of recruiting patients

Overall, 42 cases diagnosed with primary adenocarcinoma from small intestine were analyzed for clinical data, including clinical presentation, diagnostic method, pathological staging, treatment, and follow-up data. The baseline characteristics are summarized in Table 1. The median age was 54.7 years (range: 24–78 years). Twenty two (52.4%) patients were under 60 years while 27 (64.3%) patients were male. For primary symptoms at first diagnosis, 21 (50%) patients were present with intestinal obstruction, another 4 (9.5%) were diagnosed with intestinal perforation. The other 17 (40.5%) patients were present with other symptoms such as abdominal pain, abdominal masses, or melena, rather than intestinal obstruction.

**Table 1:**
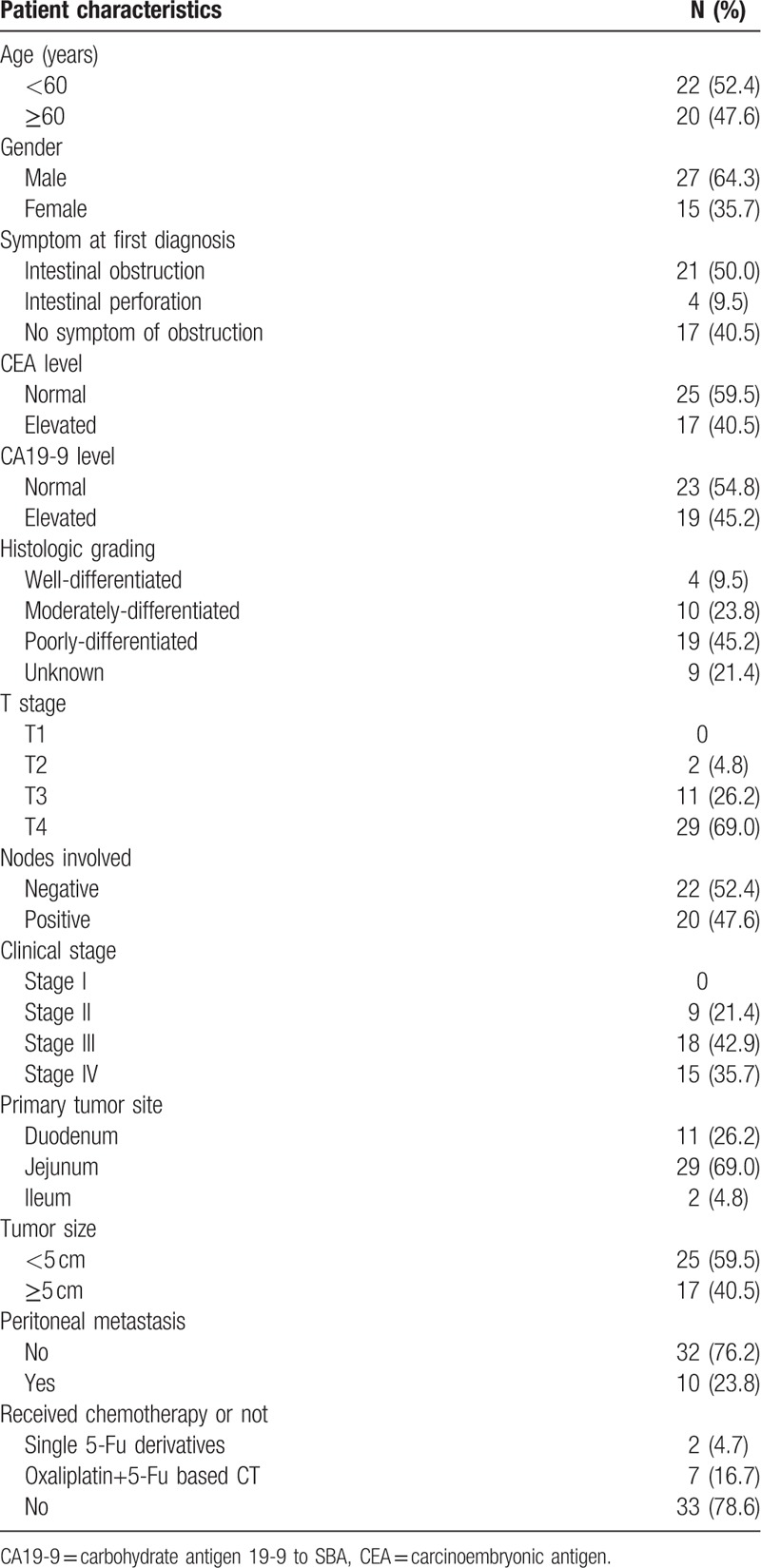
Pathological and clinical characteristics of patients with SBA.

For the levels of tumor markers, 17 (40.5%) patients had elevated carcinoembryonic antigen (CEA) level with the highest level of 600 ng/ml while 19 (45.2%) had elevated carbohydrate antigen 19-9 to SBA (CA19-9) level with the highest level of >1000 ng/ml. Among all the patients, only 3 presented with inconsistent trend between CEA and CA19-9, more specifically, 1 was with elevated CEA and normal CA19-9 while 2 were with elevated CA19-9 and normal CEA. (Table 1). The gastroduodenal endoscopy could help to discover lesions in duodenum and upper jejunum. Diagnosis of lesions distal from the segments mentioned above would have to dependent on small intestine visualization, capsule endoscopy, or enhanced abdominal CT scan. Patients that were firstly diagnosed with small intestine carcinoma by symptoms of intestinal obstruction or perforation would be dependent on emergent enhanced abdominal CT or X-ray scan followed by emergent exploratory laparotomy.

The pathological characteristics and staging information were summarized in Table 1. The histologic grading was available for 33 patients, among which 4 (9.5% out of 42) patients were well-differentiated, 10 (23.8%) were moderately-differentiated, while 19 (45.2%) were poorly-differentiated adenocarcinoma.

In terms of the TNM staging, 29 (69.0%) patients were diagnosed with T4 while another 11 (26.2%) patients were T3. Only 2 patients were diagnosed with T2, indicating early diagnosis of small intestine tumor was not easy. For the N staging, 20 (47.6%) patients were positive in node metastasis while 22 (52.4%) were negative. Total of 15 (35.7%) patients were diagnosed with distal metastasis, including 10 cases of peritoneum, 2 cases of hepatic, 2 cases of ovary, and 1 case of retroperitoneal lymph nodes metastasis. In summary, 9 (21.4%) patients were in stage II (N0, T2 to T4), 18 (42.9%) patients were in stage III (positive in lymph node metastasis), and 15 (35.7%) were in stage IV with distal metastasis. The primary sites in those patients were 11 (26.2%) from duodenum, 29 (69.0%) from jejunum, and 2 (4.8%) from ileum. The diameter of the tumors were 25 (59.5%) cases <5 cm while 17 (40.5%) cases ≥5 cm. For patients with peritoneal metastasis or not, 10 cases (23.8%) occurred tumor peritoneal metastasis.

For treatment selection of the patients, all patients in our review underwent radical resection of the primary tumor. However, among all the included patients, only 9 received adjuvant or palliative chemotherapy after the surgery, among which 2 patients received single 5-Fu derivatives, either orally or intravenously while the other 7 received oxaliplatin and 5-Fu based double-agent chemotherapy, such as XELOX or FOLFOX based chemotherapy regiment. The other 33 patients did not receive any chemotherapy, either because of their unwillingness to receive any further anti-tumor treatment, or their deteriorated physical conditions that made them lose the chance of any antitumor therapy (Table 1).

### 4.2 Patient survival time stratified by different clinical characteristics

Median OS in all patients were 24.2 months (95% CI: 4.0–72.0). Three-year and 5-year survival rate are 21% and 9%. Median OS was reviewed in different subgroups of patients (Table 2). In patients of less than 60 years old, the median survival time was 27 months (ranging from 8 to 72 months), compared to 16.5 months (ranging from 4 to 46 months) in patients with ≥60 years old, which is significantly shorter (*P* *=* .021) by univariate analysis. As for patients with intestinal obstruction at first diagnosis, the median survival was 18 months (ranging from 8 to 46 months), while patients with intestinal perforation had even worse survival as 4.5 months (ranging from 4 to 12 months). On the contrary, patients with other symptoms less severe than obstruction or perforation lived a longer time as median survival of 31 months (ranging from 10 to 72 months). Univariate analysis showed significant difference among three groups (*P* < .001).

**Table 2:**
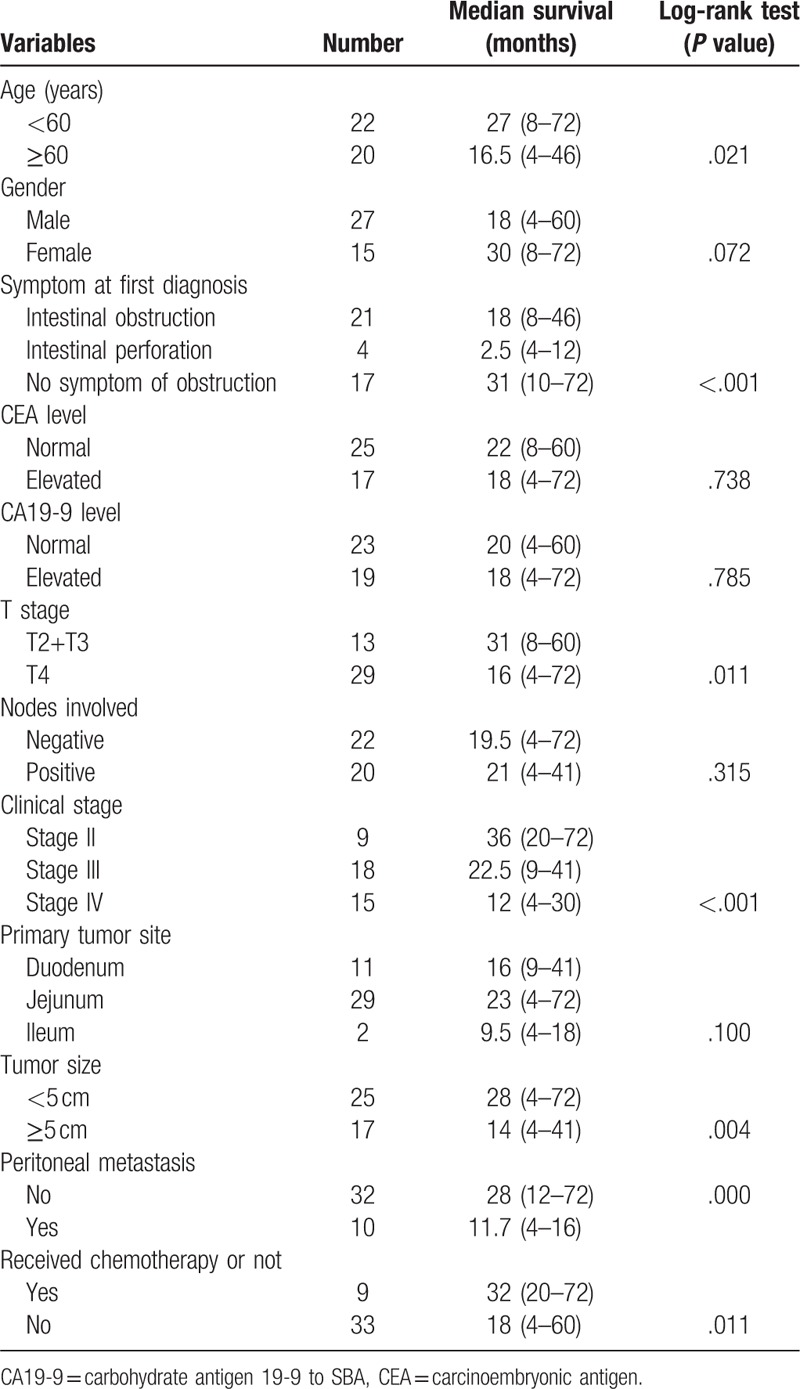
Overall survival of patients with SBA by different clinicopathological features.

**Figure 1:**
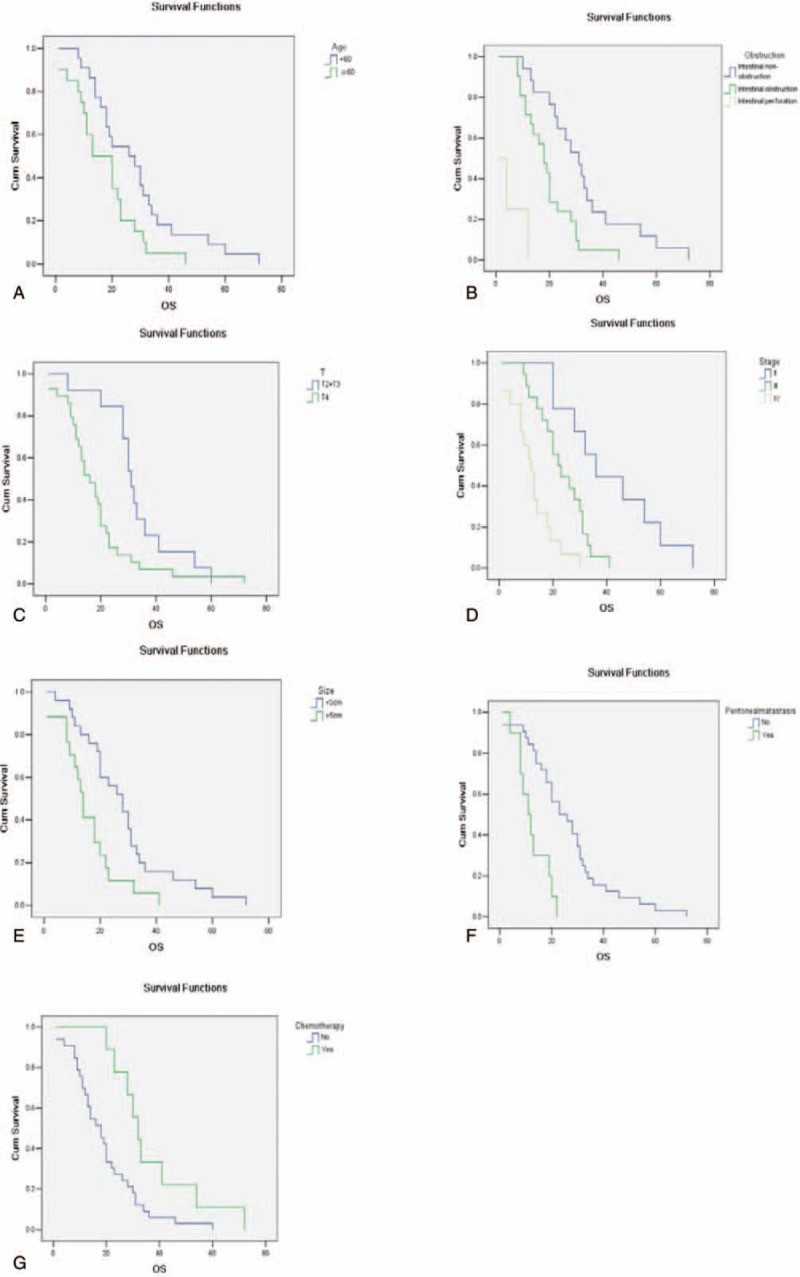
Kaplan–Meier survival curves of patients with SBA by different significant factors. (A) Kaplan–Meier survival curve for patients with ages less or more than 60 years old; (B) Kaplan–Meier survival curve for patients with different symptoms at first diagnosis; (C) Kaplan–Meier survival curve for patients with different T stages; (D) Kaplan–Meier survival curve for patients with different clinical stages; (E) Kaplan–Meier survival curve for patients with different tumor sizes; (F) Kaplan–Meier survival curve for patients with peritoneal metastasis or none; (G) Kaplan–Meier survival curve for patients with chemotherapy or not (*P* < .05).

As for the survival of patients with different staging, patients with T stage of T2 to T3 lived a median survival time of 31 months (ranging from 8 to 60 months) while patients with T4 had a median survival of only 16 months (ranging from 4 to 72 months), making the difference between groups significant (*P* *=* .011). Likewise, patients with different clinical stages lived different median survival time, as patients in stage II were 36 months (ranging from 20 to 72 months), patients in stage III were 22.5 months (ranging from 9 to 41 months) while patients in stage IV only lived a median time of 12 months (ranging from 4 to 30 months). The difference among groups was statistically significant according to univariate analysis (*P* *<* .001). However, patients with different N stages failed to present with significant difference in median survival time, as patients with negative N stage lived a median time of 19.5 months (ranging from 4 to 72 months) while patients with positive N stage lived 21 months (ranging from 4 to 41 months), with a *P* value of .315 (Fig. 1).

Other factors that might influence median survival time included primary tumor size, where patients with tumor less than 5 cm lived a median time of 28 months (ranging from 4 to 72 months) while that with tumor ≥5 cm lived only 14 months (ranging from 4 to 41 months), leaving the significant difference between groups as *P* *=* .004. Patients with peritoneal metastasis lived 28 months (ranging from 12–72 months) and those with no peritoneal metastasis lived 11.7 months (ranging from 4 to 16 months), making the difference significant (*P* < .001). Besides, patients who received chemotherapy after surgery lived a median survival time of 32 months (ranging from 20 to 72 months), while patients who did not receive any anti-tumor therapy lived a median time of only 18 months (ranging from 4 to 60 months). The difference between groups was statistical significant as *P* *=* .011.

Despite all the factors that might influence survival among patients with SBA, there were other clinical characteristics we did not find significant difference between groups, indicating those might not be predictors for patient survival, such as patient gender (*P* *=* .072), CEA or CA19-9 level (*P* *=* .738 or.785), and primary tumor site (*P* *=* .100) (Table 2)

### 4.3 Independent predictors of survival by multivariate analysis

In order to better illustrate predictors that might predict survival of patients with SBA, and eliminate interactions among factors that might influence the final results, multivariate analysis was applied. The factors analyzed in the analysis included: gender, age, initial obstruction or not, CEA level, CA19-9 level, T stage, N stage, clinical staging, primary tumor site, tumor size, peritoneal metastasis, and chemotherapy or not (Table 3). The final result showed that the *P* values including age, initial obstruction or not, clinical staging and chemotherapy or not were still statistically significant as .035, .026, .000, and .027, respectively, indicating the four factors might be true predictors of survival for patients with SBA.

**Table 3:**
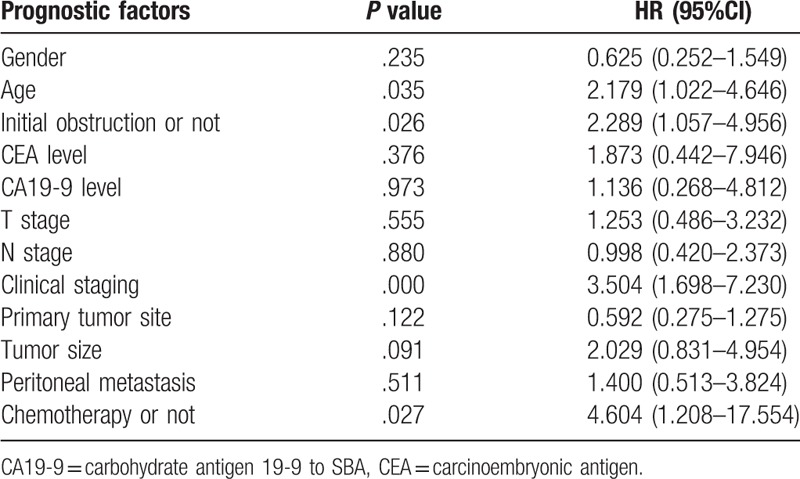
Multivariate analysis of survival in patients with small intestine adenocarcinoma.

## 5 Discussion

SBA was a relatively rare malignancy compared to gastric and colon cancer, despite that the small intestine makes up 75% of the length of the digestive tract and 90% of its mucosal surface area.^[2,12]^ In our research, patients treated in West China Hospital of Sichuan University from 2001 to 2013 were reviewed, only to find 48 cases which are definitely diagnosed with primary SBA. The average age for patients in our study was 53 years old, with male ratio higher than female (64.3% vs 35.7%), both of which were consistent with previous recordings.^[10,11,13]^ However, in 42 cases of SBA in our research, primary tumors arisen from jejunum took major part as 69%, while those from duodenum only took part as 26.2%, which was inconsistent with most research that tumor arisen from duodenum took major part in SBA.^[10,11,13]^ This might because 160 cases of tumor from duodenal periampullary orampula of vater were excluded from the research in case those tumors might be pancreatic or cholangetic originated which were hardly differentiated especially in a retrospective review.

As located deep inside the small intestine which makes early notice and diagnosis difficult, SBAs were usually diagnosed at a relatively late stage. In our research, 25 cases of patients (nearly 60%) did not have the chance to be diagnosed until intestinal obstruction or intestinal perforation finally took place. Other patients were also present with some obvious complains such as abdominal pain, abdominal mass, or melena at their first diagnosis. The routine gastroduodenoscope could only help to diagnose masses in the proximal location of small intestine, while enteroclysis of small intestine, capsule endoscopy, enteroscope or enhancement CT must be used to assist diagnosis if tumors located in distal small intestine such as jejunum or ileum. Patients with intestinal obstruction or perforation usually could not be precisely diagnosed until tumor masses were biopsied or resected during the emergent operation. In our review, most patients suffering from SBA were in a relatively late stage at the time of diagnosis, as only two patients were in T2, while patients in T3 to T4 were as much as 40, taking up more than 95% of all patients in study. Besides, only 9 patients were in clinical stage of I to II, the other 33 patients (about 80%) were in late stage as III to IV. However, when tumor size was taken into account, a relatively small portion of patients (17 patients, 40.5%) presented with tumor size ≥5 cm in diameter, which seemed to be disaccord with the fact that patients were with high proportion of intestinal obstruction or perforation at first diagnosis. Nevertheless, this seemingly self-contradictory phenomenon might indicate that it was the relatively narrower lumen of small intestine than that of large intestine, which might cause early obstruction of intestine even when the tumor was in a relatively small size, hence reminded us of the higher malignance and potential threat to patients with SBA.

On the other hand, as it comes to the tumor marker which should have been a hint for early diagnosis of malignancy, there were only about 50% of patients with SBA had elevated levels of CEA or CA19-9, which indicated that routine tests of tumor markers might not be a sensitive way of early detection for SBA. For most of the patients with SBA, it usually took a relatively long time for diagnosis and physical treatment before the final correct diagnosis. The atypical symptoms as abdominal pain, abdominal distention and changes of bowel evacuation habit, and usually negative results of routine gastroduodenoscope would easily mislead doctors to diagnose them with functional disorder of intestine, or inflammatory bowel disease, leading to delay of correct diagnosis and treatment and eventual deterioration of the disease. More confusingly, it has been confirmed that SBA were more commonly derived under some genetic predisposition,^[14,15]^ such as familial adenomatous polyposis (FAP),^[16,17]^ Lynch syndrome,^[18]^ and other predisposing conditions such as Crohn's disease,^[19,20]^ coeliac disease,^[21,22]^ etc. The predisposing conditions may cause some of the atypical symptoms, therefore make diagnosis of SBA under this condition more confusing and difficult. In order to eliminate missing diagnosis of tumor in the small bowel, one must always take precautions of patients complaining about atypical abdominal pain or distention, uncertain abdominal mass, intermittent melena or bloody stools, with negative result in gastroduodenoscope and unsatisfied effect of routine medical treatment, especially for who had previously diagnosed with predisposing genetic disorders or related diseases. Early usage of enhanced CT, enteroclysis of small intestine, enteroscope or capsule endoscopy might be necessary in case of missing diagnosis of early SBA.

According univariate analysis, the patient characteristics that might be prognostic factors of SBA were as follows: age, intestinal obstruction at first diagnosis, T4 of tumor stage, tumor size, clinical stage, peritoneal metastasis or not and whether receiving chemotherapy. Further study by multivariate analysis to eliminate possible complicated correlations between factors demonstrated that age of more than 60 years old, intestinal obstruction or perforation at first diagnosis, relatively late clinical stage, peritoneal metastasis and not receiving chemotherapy were four factors that might be related with poor prognosis of SBA. As all the cases included in this review were selected as primary tumor resected, the strongest prognostic factor as primary resection could not be revealed by this review. Other factors that might hint poor prognosis of SBA including location of tumor site as in duodenum, poorly differentiation, positive margin of resection, lymphovascular infiltration and a lymph node ratio of ≥10%^[11,23–25]^ were not revealed in this study, probably due to lack of necessary data collection by retrospective research, and relatively small cases that might mask significant differences which would have been shown in large number of samples.

In our study, we included patients with SBA that all have undergone resection of primary tumor. As is mentioned above, according to various researches before, primary resection is one of the most important factor that indicate better prognosis of SBA.^[10]^ The median survival time of patients with stage II SBA was 36 months (ranging from 20 to 72 months), that of patients with stage III SBA was 22.5 months (ranging from 9 to 41 months), who have all undergone primary resection. On the contrary, patients with stage IV SBA, despite their resection of primary tumors, only lived a median time of 12 months (ranging from 4 to 30 months) in our study, which was no longer than the median survival time reported by previous literatures.^[26–29]^ This phenomenon provided an assumption that palliative primary tumor resection might not be encouraged if there were no urgent surgical needs such as severe intestinal obstruction, perforation or medical-uncontrolled bleeding, as surgery itself might not be beneficial to improve the OS of patients with advanced SBA, which however still needs further investigation.

Although it has been proved that radical resection is beneficial for survival in patients with localized SBA, whether adjuvant chemotherapy or radiotherapy should take place after the surgery, and what should be the standard regimen still remain controversial due to lack of prospective randomized controlled trials. According to some of the retrospective researches, adjuvant chemotherapy did not improve survival in patients with localized SBA who underwent radical resection of the tumor. However, the small number of patients treated and a bias in selecting patients for treatment, or an inadequate chemotherapy regimen might be the cause of negative results.^[23,30–32]^ In 2010, Overman et al conducted a single-center, retrospective research where 54 patients with SBA underwent curative R0 resection of the tumor, followed with or without adjuvant chemotherapy were reviewed. The result showed adjuvant chemotherapy improved disease free survival (DFS) (HR 0.27; 95% CI 0.07–0.98, *P* = .05) but not OS (HR 0.47; 95% CI 0.13–1.62, *P* = .23).^[9]^ Other researches indicated that fluoropyrimidine and oxaliplatin based chemotherapy regimen might benefit patients after a curative R0 resection of a localized SBA.^[33–36]^ Based on the data above, the adjuvant fluoropyrimidine and oxaliplatin-based chemotherapy has been recommended for patients with curative R0 resection of a stage III SBA or stage II with pT4 by French guidelines (www.tncd.org, last updated in 2013).

In our study, chemotherapy was proved to benefit survival in patients with SBA. However, as the patient number in our study was small, no significant difference was observed in subgroup of patients in stage II and III treated with or without chemotherapy. Nevertheless, clear trends still provided some clues. In 6 patients with stage II who did not receive adjuvant chemotherapy after radical resection of the tumor, the median survival time was 35 months (ranging from 20 to 60 months), compared to 3 patients in the same stage who received adjuvant chemotherapy lived a median time of 52.7 months (ranging from 32 to 72 months). The regimens of chemotherapy for the 3 patients were XELOX, FOLFOX, and single fluoropyrimidine, respectively. The same trend was showed in stage III patients, where 14 patients who did not receive adjuvant chemotherapy lived a median time of 21.1 months (ranging from 9 to 34 months), while 4 patients in the chemotherapy group had a mean survival time of 30.5 months (ranging from 20 to 41 months). The chemotherapy regimens in this subgroup included 3 cases of FOLFOX, and 1 case of XELOX plus bevacizumab. This clear trend might hint us the survival benefit of adjuvant chemotherapy in localized SBA after radical resection of the tumor, however randomized trials with large number of patients are still necessary for this assumption.

The same problem also emerged in the palliative treatment of patients with advanced SBA, where prospective randomized trials with large sample of patients were also needed for the standard chemotherapy regimen. However, given the circumstances that SBA was relatively rare disease, such randomized phase III trials with large samples seems to be very difficult to be achieved, which makes results from phase II trials with high quality valuable to be considered. In 2005, a multi-institutional phase II study performed by Eastern Cooperative Oncology Group (ECOG) evaluated 39 patients with advanced SBA treated with FAM regimen, only to get a not satisfying result: the response rate was only 18.4% (95% CI 7.8–34.4), and the median survival time was 8 months.^[29]^ However, results from other phase II studies^[34,37]^ and retrospective researches^[33,38]^ indicated chemotherapy containing fluoropyrimidine and oxaliplatin seems to be the most effective regimen. In 2010, Zaanan et al conducted a retrospective multicenter study^[33]^ which evaluated LV5FU2 (n = 10), FOLFOX (n = 48), FOLFIRI (n = 19), and LV5FU2-cisplatin (n = 16) in 93 patients with advanced SBA. The median PFS times in four subgroups were 7.7, 6.9, 6.0, and 4.8 months, while the median OS times were 13.5, 17.8, 10.6, and 9.3 months, respectively, indicating the regimen of FOLFOX might be the best candidate for first line chemotherapy in patients with advanced SBA. In 2012, another multicenter retrospective study of 132 patients with unresectable SBA treated with single fluoropyrimidine, fluoropyrimidine-cisplatin, fluoropyrimidine-oxaliplatin, fluoropyrimidine-irinotecan and other regimens were taken place in Japan. The results also showed significant longest PFS (8.2 months) and substantial (but not significantly) longest OS (22.2 months) in fluoropyrimidine-oxaliplatin group.^[39]^ The oxaliplatin-based chemotherapy is therefore recommended as a first line treatment by French guidelines (www.tncd.org, last updated in 2013).

In our study, only 2 patients with stage IV SBA received palliative chemotherapy, with the regimen as LV5FU2 plus Bevacizumab, and FOLFOX plus radiotherapy of the pelvis, respectively. The survival times for the 2 patients were 23 months and 30 months, respectively, with a median survival time of 26.5 months, which is also substantially longer than the median survival time of 13 patients who did not receive any chemotherapy as 10 months (ranging from 1–19 months). The small number of cases made statistical analysis impractical, however the difference between groups still indicated the trend that chemotherapy might benefit survival in patients with advanced unresectable SBA.

When it comes to the targeted treatment of SBA, few studies could provide solid proofs. Wang et al^[40]^ analyzed EGFR mutations by immunohistochemistry (IHC) or DNA sequencing in 77 cases of patients with SBA. The results showed that mutations and amplification in EGFR genes are minor events, and most of SBAs may be unsuitable for EGFR-TKIs treatment. In 2008, Tsang et al^[41]^ reported that use of bevacizumab combined with gemcitabine and oxaliplatin in a patient with advanced unresectable SBA led to disease stabilization with a survival time of at least one year following diagnosis, compared with a mean survival time of only 8–9 months in advanced SBA treated with standard chemotherapeutical care. In our study, one patient with stage III SBA used the regimen of XELOX plus bevacizumab while another with stage IV SBA used LV5FU2 plus bevacizumab, whose survival times were 33 months and 23 months, respectively. However, as they were only single cases, statistical analysis could not be utilized for the comparison. More researches and trials on targeted therapy in advanced SBA will be needed in the future.

In order to better understand the clinical characteristics of advanced SBA, the metastatic patterns of patients with stage IV SBA were reviewed. Among the 15 patients who had distant metastasis, as much as 10 patients presented with peritoneal metastasis, while the other were found with liver (2 cases), ovary (2 cases), and retroperitoneal lymph node (1 case) metastasis, respectively. This phenomenon strongly suggested that most of the patients with advanced SBA presented with a metastatic pattern of intra-abdominal peritoneal metastasis, which significantly increased risks of intestinal adhesion, obstruction and malignant ascites that might deteriorate the physical condition of the patients. Based on this finding, whether intraperitoneal chemotherapy at a relatively early phase of advanced SBA could eliminate minute or potentially existed metasitatic nodules on peritoneum, thus improving the quality of life and decreasing the risks of related complications, should be a promising direction of research.

## 6 Conclusions

SBA was a relatively rare malignancy with relatively high chance of delayed diagnosis or misdiagnosis. Intestinal obstruction was frequently found at the first diagnosis. Peritoneal metastasis was the most popular metastatic mode for advanced SBA. Age of more than 60 years old, intestinal obstruction or perforation at first diagnosis, relatively late clinical stage, peritoneal metastasis and not receiving chemotherapy were four factors that might be related with poor prognosis of SBA. Early detection of SBA, radical resection followed by fluoropyrimidine and oxaliplatin based adjuvant chemotherapy was recommended for patients with localized SBA. Due to the high risks of peritoneal metastasis, intraperitoneal chemotherapy might play an important role in treatment of advanced SBA. Targeted therapy on advanced SBA was promising though still needs further investigations.

## Author contributions

**Conceptualization:** Dan Cao, Meng Qiu.

**Data curation:** Jiangfang Tian.

**Formal analysis:** Jiewei Liu, Yu Yang.

**Investigation:** Chunhong Guo, Jiangfang Tian,.

**Methodology:** Yu Yang.

**Resources:** Dan Cao.

**Software:** Xi Yang.

**Supervision:** Dan Cao , Hongfeng Gou.

**Writing – original draft:** Jiewei Liu, Jiangfang Tian.

**Writing – review & editing:** Dan Cao.

Jiangfang Tian orcid: 0000-0001-6082-7358.
